# Correction: Block et al. Fluorinated Analogs of Organosulfur Compounds from Garlic (*Allium sativum*): Synthesis, Chemistry and Anti-Angiogenesis and Antithrombotic Studies. *Molecules* 2017, *22*, 2081

**DOI:** 10.3390/molecules28031025

**Published:** 2023-01-19

**Authors:** Eric Block, Benjamin Bechand, Sivaji Gundala, Abith Vattekkatte, Kai Wang, Shaymaa S. Mousa, Kavitha Godugu, Murat Yalcin, Shaker A. Mousa

**Affiliations:** 1 Department of Chemistry, University at Albany, State University of New York, Albany, NY 12222, USA; 2 The Pharmaceutical Research Institute, Albany College of Pharmacy and Health Sciences, Rensselaer, NY 12144, USA; 3 Department of Physiology, Veterinary Medicine Faculty, Uludag University, Bursa 16059, Turkey

In the original publication [1], there were mistakes in the representative images for the PBS and FGF controls and FGF + Difluoroallicin (Figure 5). Specifically, in Figure 5, there were unintentional mistakes in incorporating representative CAM images from other archived files. Additionally, the error in the FGF + Difluoroallicin panel was due to inadvertent exchange with a panel from another contemporaneous study. The correct image for those three panels (PBS, FGF and FGF + Difluoroallicin) appears in the corrected figure. The authors apologize for these unintentional mistakes. However, the mean % inhibition of FGF-mediated angiogenesis by difluoroallicin (>60% inhibition) was unaffected, as shown in Figure 3. These changes in the representative images do not affect the conclusions regarding the biological activity of the compounds studied.

Figure 5 (Corrected):

**Figure 5:**
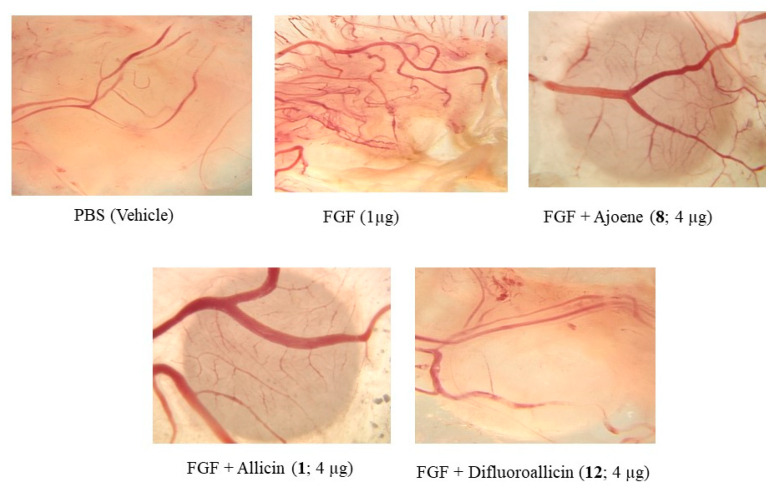
Representative images of CAM neovascularization induced by FGF (bFGF) and its inhibition by the various garlic-derived compounds, including ajoene (**8**), allicin (**1**) and difluoroallicin (**12**) **each at** 4 µg/20 µL/CAM. The images shown are representative single images selected for illustration of the general anti-angiogenesis efficacy of the organosulfur compounds and not for quantitative purposes.

This correction was approved by the Academic Editor. The original publication has also been updated.
